# Nrf2 Signaling Pathway in Chemoprotection and Doxorubicin Resistance: Potential Application in Drug Discovery

**DOI:** 10.3390/antiox10030349

**Published:** 2021-02-26

**Authors:** Sepideh Mirzaei, Ali Zarrabi, Farid Hashemi, Amirhossein Zabolian, Hossein Saleki, Negar Azami, Soodeh Hamzehlou, Mahdi Vasheghani Farahani, Kiavash Hushmandi, Milad Ashrafizadeh, Haroon Khan, Alan Prem Kumar

**Affiliations:** 1 Department of Biology, Faculty of Science, Islamic Azad University, Science and Research Branch, Tehran 1477893855, Iran; sepidehmirzaei.smv@gmail.com; 2 Sabanci University Nanotechnology Research and Application Center (SUNUM), Tuzla 34956, Istanbul, Turkey; alizarrabi@sabanciuniv.edu (A.Z.); milad.ashrafizadeh@sabanciuniv.edu (M.A.); 3 Department of Comparative Biosciences, Faculty of Veterinary Medicine, University of Tehran, Tehran 1417466191, Iran; faridhashemi172@gmail.com; 4 Young Researchers and Elite Club, Tehran Medical Sciences, Islamic Azad University, Tehran 1477893855, Iran; ah_zabolian@student.iautmu.ac.ir (A.Z.); h.saleki@student.iautmu.ac.ir (H.S.); n.azami@student.iautmu.ac.ir (N.A.); ss.hamzehlou@gmail.com (S.H.); Mahdi.vf.1997@gmail.com (M.V.F.); 5 Department of Food Hygiene and Quality Control, Division of Epidemiology, Faculty of Veterinary Medicine, University of Tehran, Tehran 1417466191, Iran; houshmandi.kia7@ut.ac.ir; 6 Faculty of Engineering and Natural Sciences, Sabanci University, Orta Mahalle, Üniversite Caddesi No. 27, Orhanlı, Tuzla 34956, Istanbul, Turkey; 7 Department of Pharmacy, Abdul Wali Khan University, Mardan 23200, Pakistan; haroonkhan@awkum.edu.pk; 8 Cancer Science Institute of Singapore, Department of Pharmacology, Yong Loo Lin School of Medicine, National University of Singapore, Singapore 117599, Singapore; 9 NUS Centre for Cancer Research, Yong Loo Lin School of Medicine, National University of Singapore, Singapore 117597, Singapore

**Keywords:** doxorubicin, chemoresistance, oxidative stress, redox signaling, nuclear factor erythroid 2-related factor 2 (Nrf2), cancer therapy

## Abstract

Doxorubicin (DOX) is extensively applied in cancer therapy due to its efficacy in suppressing cancer progression and inducing apoptosis. After its discovery, this chemotherapeutic agent has been frequently used for cancer therapy, leading to chemoresistance. Due to dose-dependent toxicity, high concentrations of DOX cannot be administered to cancer patients. Therefore, experiments have been directed towards revealing underlying mechanisms responsible for DOX resistance and ameliorating its adverse effects. Nuclear factor erythroid 2-related factor 2 (Nrf2) signaling is activated to increase levels of reactive oxygen species (ROS) in cells to protect them against oxidative stress. It has been reported that Nrf2 activation is associated with drug resistance. In cells exposed to DOX, stimulation of Nrf2 signaling protects cells against cell death. Various upstream mediators regulate Nrf2 in DOX resistance. Strategies, both pharmacological and genetic interventions, have been applied for reversing DOX resistance. However, Nrf2 induction is of importance for alleviating side effects of DOX. Pharmacological agents with naturally occurring compounds as the most common have been used for inducing Nrf2 signaling in DOX amelioration. Furthermore, signaling networks in which Nrf2 is a key player for protection against DOX adverse effects have been revealed and are discussed in the current review.

## 1. Introduction

Doxorubicin (DOX) is an anthracycline isolated from Streptomyces with proficiency in treatment of various cancers such as thoracic cancers, reproductive cancers, gastrointestinal and brain tumors [1]. Three major mechanisms are followed by DOX in suppressing progression and proliferation of cancer cells, including inhibiting DNA topoisomerase II activity, DNA intercalation and enhancing production of free radicals, especially reactive oxygen species (ROS) that are of importance in triggering apoptosis through mitochondrial pathway [2]. After its discovery, DOX was considered as the first option in treatment of cancer patients and showed promising clinical results. However, these ideal findings disappeared with development of DOX resistance [3,4,5,6].

Currently, two major obstacles are considered for cancer chemotherapy with DOX including A) DOX resistance, and B) dose-dependent toxicity [7,8,9]. The toxicity of DOX against normal cells has a negative impact on its efficacy in cancer therapy, since high dose of DOX cannot be administered to cancer patients to overcome resistance. In order to reverse DOX resistance, a combination of DOX with other compounds such as selenium is utilized to induce apoptosis and necrosis in cancer cells, leading to their enhanced sensitivity to DOX chemotherapy [10]. Mitochondrial transcription factor A (TFAM) stimulates mitochondrial dysfunction and AMP-activated protein kinase (AMPK) in suppressing DOX resistance [11]. That is why molecular pathways that promote cancer cell growth and viability, can induce DOX resistance. For instance, in non-small cell lung cancer, vasohibin2 (VASH2) functions as a tumor-promoting factor in enhancing proliferation that subsequently, stimulates DOX resistance [12]. Identification of such factors is of importance in suppressing DOX resistance by developing potential therapeutics for their targeting [13].

One of the processes contributing to DOX resistance is glycolysis. Cancer cells demonstrate enhanced glucose uptake and, to have enough energy, they induce glycolysis as a way of reaching a high amount of energy in a low time. In osteosarcoma cells, sphingosine kinase 1 (Sphk1) undergoes up-regulation due to hypoxia and activation of hypoxia-related molecular pathway, known as hypoxia-inducible factor 1α (HIF-1α) [14,15]. Then, an increase occurs in glycolysis, providing condition for DOX resistance [16]. Non-coding RNAs (ncRNAs) such as long non-coding RNAs (lncRNAs) and microRNAs (miRNAs) also participate in development of DOX resistance due to their regulatory effects on biological mechanisms and molecular pathways [17,18,19,20,21]. In addition to recognition of tumor-promoting molecular pathways and using combination chemotherapy, another strategy that utilizes nanostructures for DOX delivery has been developed. This strategy is ideal for in vitro and in vivo experiments and nanoparticles can provide a platform for co-delivery of DOX with other anti-tumor agents, leading to targeted delivery at tumor site and reversing chemoresistance [22]. Therefore, the DOX resistance is an increasing challenge, and more experiments are required to find novel strategies in reversing chemoresistance.

Another obstacle in using DOX in cancer chemotherapy is its dose-dependent toxicity. Clinical studies have confirmed this issue. Cardiomyopathy, gastrointestinal (GI) side effects and hematological abnormalities result from using DOX alone or in combination with other chemotherapeutic agents in cancer therapy [23,24,25]. Increased level of oxidative stress and subsequent apoptosis induction are responsible for DOX toxicity [26]. Furthermore, DOX can stimulate matrix metalloproteinase-2 (MMP-2) for mediating cardiotoxicity. Application of MMP inhibitors is associated with inhibiting intracellular and extracellular matrix remodeling and ameliorating DOX toxicity [27]. What is noteworthy is that a number of plant-derived natural compounds such as alpha-tocopheryl succinate [28], naringenin [29] and atorvastatin [30] have been applied in DOX side effect alleviation. These compounds mainly diminish oxidative stress, inflammation and apoptosis.

These studies demonstrate that free radical generation is the most important way that DOX follows in cancer therapy. However, free radicals can negatively affect major organs in the body such as kidney, liver and brain. In the present review, we focus on a molecular pathway which involves nuclear factor erythroid 2-related factor 2 (Nrf2) as a regulator of oxidative stress in cells. Although activation of Nrf2 signaling protects cells against oxidative damage [31], it can induce chemoresistance via suppressing oxidative-mediated cell death in cancer cells [32].

## 2. Materials and Methods

In searching and collecting data for the current review, we used databases such as Pubmed, Google Scholar and Science Direct. Keywords such as “Nrf2 + Doxorubicin”, “Nrf2 + resistance”, and “Nrf2 + chemoprotection” were used. Furthermore, most of the experiments and articles are from 2020.

## 3. Nrf2 Signaling Pathway

Counteracting oxidative stress and inflammation is the main aim of Nrf2 signaling in cell protection [33,34,35,36,37,38,39,40,41,42,43,44]. Sequestration of Nrf2 occurs in normal conditions by Kelch-like ECH-associated protein 1 (Keap1), when ROS and oxidative levels are at standard limit [45]. For providing proteasomal degradation of Nrf2, preventing its accumulation in cytoplasm and subsequent translocation to nucleus, Keap1 as a ubiquitin ligase adaptor protein, represents Nrf2 to Cullin-3 (Cul3)/RBX1 complex [46]. In contrast, electrophiles and oxidative stress are considered as inducers of Nrf2 signaling. In this way, Keap1 dissociation from Cul3 occurs via structural modification of Keap1 at cysteine 151 [47]. Furthermore, glycogen synthase kinase-3β (GSK-3β) prevents Nrf2 degradation by Nrf2 phosphorylation at serine 335 and 338. Then, Nrf2 polyubiquitination and its identification by β transducin repeat containing E3 ubiquitin-protein ligase (βTrCP) occur that are in favor of preventing Nrf2 degradation and providing CUL3/RBX1-induced degradation [48,49].

**Figure 1:**
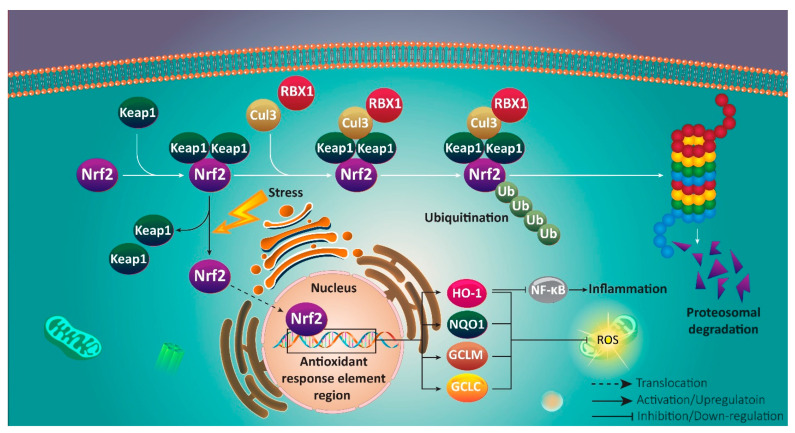
A schematic presentation of Nrf2 signaling pathway. Oxidative stress induces nuclear translocation of Nrf2 to promote antioxidant activity via up-regulating HO-1, NQO1, and GCLM.

As a result, high levels of Nrf2 accumulate in cytoplasm that is followed by nuclear translocation and targeting genes containing antioxidant response element (ARE) region [50]. These genes include heme oxygenase-1 (HO-1), NAD(P)H dehydrogenase quinone 1 (NQO1), *γ*-glutamyl cysteine ligase modulatory and catalytic subunits (GCLM and GCLC, respectively), and ferritin accounting for inducing oxidant and antioxidant balance in cells [51,52,53,54,55]. Noteworthy, Nrf2 activation can be beneficial in reducing inflammation via activating HO-1, and subsequent inhibition of NF-κB signaling, which generally acts as a tumor-promoting factor [56,57,58]. Nrf2 down-regulation is associated with an increase in inflammatory response via NF-κB activation [59]. Furthermore, it has been reported that Nrf2 activation is in favor of reducing levels of pro-inflammatory cytokines in cells (Figure 1) [60,61,62,63,64,65].

## 4. Nrf2 in Protection and Chemoresistance

Chemoresistance remains a major challenge for cancer therapy [66,67,68,69,70]. The dual role of Nrf2 during cancer chemotherapy has been investigated in a variety of experiments. First, activation of Nrf2 signaling is advantageous in reducing side effects of chemotherapeutic agents. For instance, paclitaxel exposure is associated with induction of mechanical allodynia, while stimulation of Nrf2 signaling by oltipraz significantly reduces this adverse impact via HO-1 induction [71]. Furthermore, peroxisome proliferator-activated receptor gamma (PPARγ) can function as an upstream inducer of Nrf2 signaling in alleviation of paclitaxel-induced mechanical allodynia [72]. Exposing cells to cisplatin enhances levels of oxidant parameters such as malondialdehyde (MDA) and reduces activity and levels of antioxidant enzymes such as superoxide dismutase (SOD), catalase (CAT) and glutathione (GSH). Nrf2, as a cytoprotective mechanism, supports kidney cells against oxidative stress and apoptosis via reinforcing antioxidant defense system [73]. Furthermore, Nrf2 activation is of importance in reducing cisplatin-mediated toxicity in reproductive system. In this way, tadalafil diminishes apoptosis and oxidative stress via Nrf2 up-regulation [74]. It has been reported that activation of Nrf2/HO-1 signaling is in favor of enhancing cell survival upon chemotherapy [75]. What is noteworthy is that phytochemicals such as curcumin [76] and formononetin [77] induce Nrf2/HO-1 signaling in reducing toxicity of oxaliplatin against liver and brain cells. These studies clearly demonstrate that Nrf2 signaling is of importance for alleviation of chemotherapy-mediated side effects.

Although Nrf2 signaling activation is of interest in reducing chemotherapy-mediated side effects, increasing evidence demonstrates association of Nrf2 activation with chemoresistance. Tumor-promoting factors such as bone morphogenetic proteins (BMP) induce Nrf2 signaling in promoting cancer cells survival and triggering chemoresistance [78]. It seems that Nrf2 can enhance tumor-initiating cell lineage that subsequently, mediates chemoresistance [79]. The p53 can provide proteasomal degradation of Keap1 via inducing Nrf2/ARE signaling to promote proliferation and apoptosis inhibition, resulting in chemoresistance [80]. Upon Nrf2 activation, glutamine metabolism increases to induce chemoresistance, and is associated with poor prognosis of cancer patients [81]. Furthermore, Nrf2 can positively interact with TAZ member of Hippo signaling in providing chemoresistance [82]. Anti-tumor compounds such as ailanthone [83] and kaempferol [84] decrease Nrf2 expression in promoting oxidative damage and ROS levels as well as triggering apoptosis, leading to enhanced cancer sensitivity to chemotherapy. Overall, studies are in agreement with the fact that Nrf2 activation induces chemoresistance [85] and its inhibition can be considered as an ideal strategy in reversing drug resistance.

## 5. Natural Compounds in Ameliorating Doxorubicin-Mediated Toxicity

As it was discussed earlier, plant derived-natural compounds are able to regulate Nrf2 signaling in exerting their protective effect against oxidative stress-mediated diseases [86,87,88]. Pristimerin (Pris) is a natural triterpenoid compound derived from Celastraceae plant with different pharmacological activities such as anti-tumor, anti-inflammatory and antioxidant [89,90]. In respect of the potential of Pris in regulating Nrf2 signaling, it can be beneficial in ameliorating DOX-mediated cardiotoxicity that could be developed due to increased oxidative stress and ROS levels as the main risk factors. In this way, Pris enhances expression of Nrf2 at mRNA and protein levels, resulting in an increase in expression of its downstream targets including NQO1, HO-1 and GCL. Then, oxidative stress parameters undergo a decrease, while antioxidant defense system is reinforced, leading to decreased DOX-mediated cardiotoxicity [91]. In addition to heart, kidney and liver are negatively affected following DOX administration due to an increase in oxidative stress and inflammation [92,93]. Asiatic acid (AA) is another phytochemical that has been under attention due to its efficacy in preventing ageing, improving wound healing and exerting anti-tumor activity [94,95,96,97]. Recently, it has been shown that AA possesses high antioxidant potential that is of importance in reducing toxic effects of DOX against major organs of body. For this purpose, AA increases Nrf2 expression that diminishes necrosis, hyaline degeneration and congestion in heart. Hepatoprotective effects include reducing leukocyte inflammation, necrosis and apoptosis. Finally, kidney is protected against DOX toxicity via decreasing necrosis and inflammation [98]. These studies reveal that Nrf2 not only protects cells against DOX-mediated oxidative stress, but also decreases inflammation, and is therefore responsible for reducing cell death.

AMPK is considered as upstream mediator of Nrf2 signaling. It seems that AMPK activation is vital for inducing Nrf2 signaling [99]. By stimulating Nrf2 signaling, AMPK protects against oxidative stress and enhances expression of downstream targets such as HO-1 [100]. β-LAPachone (B-LAP) as a protective agent, targets AMPK/Nrf2 signaling in reducing DOX-mediated cardiotoxicity. B-LAP promotes expression of AMPK to induce Nrf2 signaling for elevating expression levels of SOX, CAT and GPX, leading to amelioration of DOX-mediated cardiotoxicity [101]. Following Nrf2 activation by phytochemicals, ROS levels decrease, preventing mitochondrial dysfunction and subsequent induction of apoptosis in cells exposed to DOX [102].

Cardamonin (CAR), a flavone exclusively found in Alpinia plant, has demonstrated potential in reducing oxidative stress via regulating Nrf2 signaling. It is noteworthy that CAR activates Nrf2 signaling that is of importance in reducing Th2 cytokine generation and preventing dermatitis [103]. In vivo experiment on mice demonstrates that CAR alleviates myocardial contractile dysfunction via enhancing Nrf2 expression [104]. These studies reveal that Nrf2 is a potential target of CAR in cell protection, and similarly, CAR follows a same pathway in reducing DOX-mediated toxicity. Both inflammation and oxidative stress are inhibited by CAR administration. This is mediated by activating Nrf2 signaling and subsequent up-regulation of SOD, GSH, CAT and reduced levels of ROS [105]. Chitosan oligosaccharide (COS) is a hydrolyzed form of chitosan that is found in exoskeleton of crustaceans and walls of fungi and insects [106]. A wide variety of biological activities including immune response regulation, anti-tumor, antimicrobial and anti-apoptosis are considered for COS [107,108,109,110,111]. A recent study has shown that COS can prevent oxidative damage and induce heart growth upon exposure to DOX. COS reduces ROS levels, mitochondrial dysfunction and apoptosis in cells. Mechanistically, COS induces AMPK in triggering Nrf2/ARE axis [112]. This study demonstrates that complicated signaling networks are involved in protecting against DOX-mediated toxicity in which Nrf2 signaling is the key player. The aim of Nrf2 activation is to up-regulate expression of downstream targets such as HO-1 and NQO1 that participate in improving antioxidant/oxidant balance and ameliorating DOX-mediated toxicity [113,114].

The *p*-coumaric acid (*p*CA) is a phenolic compound that functions as a ROS scavenger in reducing oxidative stress and protecting cells against drug toxicity [115]. Different molecular pathways are affected by *p*CA in exerting its protective effects and Nrf2 is among them. In this way, *p*CA promotes Nrf2 expression to prevent ROS generation and inflammation caused by lipopolysaccharide (LPS) [116]. It seems that *p*CA inhibits acute lung injury via AMPK/Nrf2/HO-1 axis activation to induce antioxidant response [117]. These studies advocate the fact that *p*CA has potential modulatory effects on Nrf2 signaling. *p*CA significantly increases cell survival and prevents apoptosis via caspase-3 down-regulation. It has been reported that ROS generation inhibition and preventing mitochondrial dysfunction are major mechanisms for protecting against DOX-mediated cardiotoxicity. Consequently, *p*CA induces Nrf2 signaling to inhibit ROS overgeneration, preventing subsequent mechanisms that are essential for DOX-mediated cardiotoxicity [118]. Therefore, using compounds inducing Nrf2 signaling can protect against DOX-mediated cardiotoxicity [119].

Tanshinone IIA (Tan IIA) is a potent antioxidant agent exclusively found in Radix *Salvia miltiorrhiza* [120]. Similar to other phytochemicals with antioxidant activity, Tan IIA targets Nrf2 signaling. Tan IIA administration is associated with improvement in silica-mediated pulmonary inflammatory response, structural damage and fibrosis via Nrf2/ARE activation [121]. Furthermore, Tan IIA prevents liver injury via epigenetic activation of Nrf2 and reinforcing antioxidant defense system [122]. Hence, Tan IIA stimulates Nrf2 signaling as a way of recovering redox homeostasis and inhibiting pulmonary fibrosis [123]. In alleviation of DOX-induced cardiotoxicity, Tan IIA increases cell viability and prevents damage-associated morphological alterations in H9C2 cells. In addition, a decrease occurs in generation of ROS levels, while GSH undergoes up-regulation in activity. These protective effects of Tan IIA are mediated by activating Nrf2 signaling and its downstream targets HO-1 and NQO1 [124].

Punicalagin (PUN) is a polyphenol isolated from pomegranate and displays a variety of pharmacological activities, of which antioxidant and anti-inflammatory are the most important [125,126]. It seems that antioxidant activity of PUN is mediated via its impact on Nrf2 signaling pathway. In this way, PUN induces Nrf2/HO-1 axis in protecting DOX-mediated cardiotoxicity. The protective impacts of PUN are abolished via Nrf2 down-regulation. By Nrf2 activation, PUN not only reduces oxidative stress parameters, but also prevents loss of mitochondrial membrane potential, cytochrome C release and apoptosis induction [127]. Experiments discussed in this section demonstrate that phytochemicals can effectively induce Nrf2 signaling in protecting against DOX-mediated toxicity.

Importantly, as natural compounds suffer from poor bioavailability, using nanoparticles for their delivery can promote their therapeutic effects and impact on Nrf2 signaling that are of importance for ameliorating DOX-mediated toxicity. Future studies will shed some light on this aspect.

## 6. Nrf2 Modulation

MiRNAs are upstream mediators of a variety of molecular pathways due to their role in coordinating detailed biological mechanisms [128,129,130,131,132,133,134,135]. As occurs in different pathological events, miRNA dysregulation leads to alterations in normal cellular events [136,137]. Increasing evidence demonstrates that Nrf2 signaling is under surveillance of miRNAs in different pathological events for regulating response of cells to oxidative stress [138,139]. It is noteworthy that miRNA and Nrf2 interaction is of importance in DOX toxicity. It has been reported that miRNA-140-5p binds to 3^/^-untranslated region (3^/^-UTR) to reduce its expression. This leads to a reduction in activity of antioxidant enzymes such as HO-1, NQO1, and GCLM that subsequently, deteriorates DOX-mediated cardiotoxicity [140]. MiRNA-200a is another non-coding RNA that regulates Nrf2 signaling. It appears that miRNA-200a ameliorates diabetes endothelial dysfunction via reducing Keap1 expression and subsequent induction of Nrf2 signaling [141]. Furthermore, miRNA-200a inhibits apoptosis and inflammation in cardiomyocytes by regulating Keap1/Nrf2 signaling in favor of cell protection [142]. In mice exposed to DOX, miRNA-200a enhances Nrf2 expression to improve contractile function, and prevent apoptosis and oxidative stress [143].

The involvement of Nrf2 signaling in organ protection is confirmed by the study of Li and colleagues showing that Nrf2 silencing deteriorates DOX-mediated toxicity [144]. This experiment demonstrated a novel pathway in which Nrf2 follows to alleviate DOX-mediated cardiotoxicity. In this way, Nrf2 affects a mechanism known as autophagy. Primarily, autophagy is considered as a “self-digestion” mechanism that degrades toxic and aged organelles and macromolecules [145,146]. It has been reported that there is a close association between autophagy and oxidative stress in cells, so that autophagy activation can ameliorate oxidative stress [147]. This relationship is of importance in relieving oxidative damage in cells. For instance, autophagy stimulation can improve integrity of intestinal barrier against ROS and oxidative damage [148]. In reducing DOX-mediated cardiac dysfunction, Nrf2 activation can promote levels of light chain-3II (LC-3II) to induce autophagy, leading to amelioration of DOX-mediated cardiotoxicity [144]. Following Nrf2 degradation and inhibition, oxidative stress increases and apoptosis markers such as caspase-3 and caspase-9 undergo up-regulation that mediate toxic effects of DOX on organs of body [149]. An interesting point should be noted that autophagy has also interactions with apoptosis [150,151,152]. In respect to effect of Nrf2 on autophagy and also, the interaction between autophagy and apoptosis, further studies can evaluate autophagy induction by Nrf2, and its impact on incidence of apoptosis in cells exposed to DOX.

Orosomucoid 1 (ORM1) was first discovered by Tokita and Schmid in a century ago and is an acute phase protein synthesized in liver [153,154]. ORM1 has a variety of functions in cells including modulating immune system, preserving capillary barrier function, and reducing ROS levels [155,156,157]. Due to its impact of oxidative stress and ROS levels, ORM1 may be capable of regulating Nrf2 signaling. It has been reported that ORM1 overexpression is associated with activation on Nrf2 signaling and its downstream target HO-1, leading to a reduction in oxidative stress and apoptosis (caspase-3 down-regulation) that are of importance in ameliorating DOX-mediated cardiotoxicity [158].

**Figure 2:**
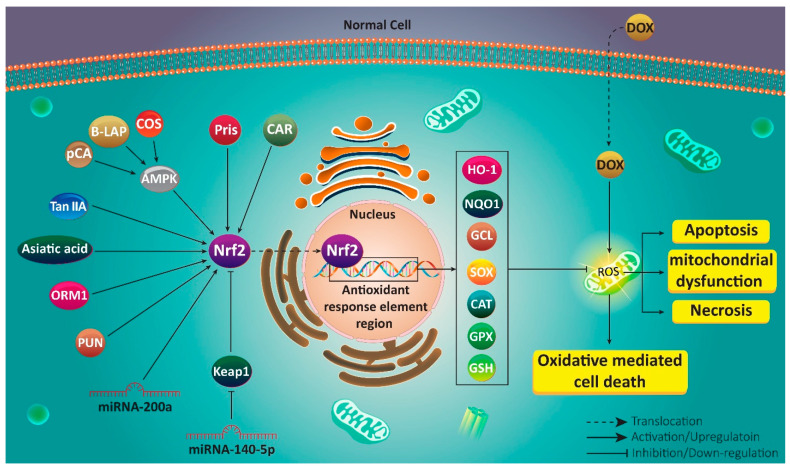
Targeting Nrf2 signaling pathway in chemoprotection. Regulation of Nrf2 signaling by upstream mediators and protective compounds in decreasing adverse effects of doxorubicin. Apoptosis, mitochondrial dysfunction, necrosis and cell death are prevented upon Nrf2 activation.

A 3-dimensional model (3D) of cardiac system demonstrates that Nrf2 activation is a positive factor in protecting cells against DOX-mediated cardiotoxicity, further confirming role of Nrf2 in cardioprotection [159]. One of the emerging upstream mediators of Nrf2 signaling is GSK-3β that is a serine/threonine kinase with ubiquitous expression [160,161]. GSK-3β is an inhibitor of Nrf2 signaling and is correlated with development of a variety of pathological events including diabetes [162], aging [163], liver disease [164] and neurological disorders [165,166,167]. There is a reverse relationship between Nrf2 signaling and GSK-3β in cells exposed to DOX, so that GSK-3β down-regulation provides condition for Nrf2 activation and reducing DOX-mediated toxicity [168]. It is worth mentioning that chronic exposure to DOX is associated with Nrf2 inhibition that further aggravates organ toxicity [169]. Therefore, a wide variety of signaling networks, both inhibitor and inducer of Nrf2 signaling are involved in regulated DOX-mediated toxicity on organs and understanding their interactions can provide a new insight for designing novel therapeutics (Table 1, Figure 2).

## 7. Nrf2 in Doxorubicin Resistance

Due to the potential role of Nrf2 signaling in triggering DOX resistance, much attention has been directed towards targeting this pathway in reversing chemoresistance. For this purpose, Singh and colleagues have designed a small molecule inhibitor of Nrf2 signaling, known as ML385 that binds to Neh1 domain of Nrf2 and inhibits its DNA binding. This leads to an increase in anti-tumor activity of DOX against lung cancer cells [172]. Therefore, first strategy can be considered developing a novel inhibitor capable of binding to Nrf2 domain and suppressing its activity and nuclear translocation. The second strategy that has not been tried yet, but can be considered in next experiments, is designing molecules capable of binding to Keap1 and promoting its activity in inhibiting Nrf2. Furthermore, natural products can be utilized in targeting Nrf2 signaling for providing DOX sensitivity. Parthenolide (PN) is a sesquiterpene lactone exclusively found in *Tanacetum parthenium* and is famous due to its inhibitory effect on cancer progression [173,174,175]. In DOX-resistance breast cancer cells, Nrf2 undergoes up-regulation that mediates increased levels of P-glycoprotein (P-gp), Bcl-2, CAT, SOD and heat shock protein 70 (HSP70). PN administration along with DOX inhibits Nrf2 signaling and its downstream targets to promote ROS generation, leading to reversing DOX resistance [176].

Chrysin is a flavonoid compound that has demonstrated anti-tumor activity against different cancers via regulating molecular pathways. Increasing evidence exhibits that chrysin suppresses cancer progression and proliferation and stimulates apoptosis via down-regulating phosphoinositide 3-kinase (PI3K)/protein kinase B (Akt) axis [177,178]. Furthermore, chrysin diminishes expression level of extracellular-signal regulated kinase (ERK) in disrupting cancer progression [179,180]. In enhancing DOX sensitivity of cancer cells, chrysin affects two distinct pathways including PI3K/Akt/Nrf2 and ERK/Nrf2. In this way, chrysin reduces expression levels of ERK and PI3K/Akt to suppress Nrf2 signaling, leading to enhanced DOX sensitivity [181]. What is noteworthy is that it seems that PI3K/Nrf2 signaling has association with activity and expression of drug resistance proteins that has been evaluated. Vialenin P (VP) can suppress PI3K/Nrf2 signaling to down-regulate multidrug resistance protein 1 (MRP1), leading to enhanced accumulation of DOX in breast cancer cells, and sensitizing them to chemotherapy [182]. 

Apigenin (APG) is a natural bioflavonoid present in fruits and vegetables and has anti-tumor activity against different cancers. APG administration is of importance in suppressing cisplatin resistance of ovarian cancer cells via apoptosis induction and Mcl-1 down-regulation [183]. In increasing DOX cytotoxicity, APG inhibits DNA repair of breast cancer cells [184]. Furthermore, co-administration of APG with other anti-tumor agents such as sorafenib increases its efficacy in triggering apoptosis and cell cycle arrest in cancer cells [185]. In DOX-resistant hepatocellular carcinoma cells, APG enhances expression of miRNA-101 as a tumor-suppressing factor. Then, up-regulated miRNA-101 reduces Nrf2 expression to promote DOX sensitivity of cancer cells [186].

In addition to miRNAs, PI3K/Akt signaling pathway is affected by APG in suppressing DOX resistance. As it was mentioned, PI3K/Akt induction is in favor of cancer progression and its inhibition can be considered as a promising strategy in cancer therapy [187,188]. It seems that APG down-regulates PI3K/Akt signaling to inhibit Nrf2, leading to increased sensitivity to DOX chemotherapy [189]. These studies demonstrate that how molecular pathways such as Nrf2 with its main role in providing redox balance, can participate in DOX resistance [190]. Consequently, targeting Nrf2, and its upstream and downstream mediators can be considered as ideal strategies in cancer therapy and reversing DOX resistance [191].

Wogonin is a bioactive flavonoid isolated from *Scutellaria baicalensis* with capability of suppressing chemoresistance via down-regulating expression and activity of P-gp [192]. Wogonin functions as an enhancer of ROS levels in inducing cell proliferation inhibition [193]. Wogonin can enhance efficacy of immune system in cancer eradication and promote macrophage M1 polarization [194]. In DOX-resistant breast cancer cells, wogonin suppresses defense system mediated via Nrf2 inhibition and decreasing expressions of HO-1 and NQO1 [195]. Another experiment investigates potential of plant-derived extracts in increasing sensitivity of lung cancer cells to DOX chemotherapy. It has been reported that cinnamomic cortex extract provides Nrf2 down-regulation in promoting DOX sensitivity [196].

Similar to wogonin, luteolin also belongs to flavonoid family. Luteolin acts as a potent anti-cancer agent [197] in suppressing cancer migration and invasion via down-regulating epithelial-to-mesenchymal transition (EMT) and focal adhesion kinase [198,199]. Cancer progression occurs in hypoxic conditions, and luteolin administration is of importance in reducing HIF-1α expression and disrupting hypoxia-mediated cancer progression. Furthermore, luteolin induces apoptosis and autophagy in breast and colon cancer cells [200]. These studies advocate the fact that luteolin administration negatively affects cancer progression, and this agent is advantageous in reversing DOX resistance. In this way, luteolin reduces Nrf2 expression at mRNA level by 34% and is involved in regaining sensitivity of lung cancer cells to DOX chemotherapy [201]. Luteolin can also enhance DOX sensitivity of breast cancer cells. Luteolin dually inhibits Nrf2/HO-1 and Nrf2/MDR1 signaling pathways to remove defense system mechanism in enhancing DOX sensitivity [202].

Consequently, using anti-tumor compounds, as most of them are phytochemicals, is of importance in reversing DOX resistance via Nrf2 down-regulation [203]. However, it should be noted that anti-tumor agents, especially naturally occurring compounds suffer from poor bioavailability [204,205], and using carriers such as nanostructures for their delivery can remarkably promote their potential in down-regulating Nrf2 signaling and enhancing DOX sensitivity of cancer cells.

## 8. Nrf2, Upstream and Downstream Targets

In the previous section, we demonstrated that both synthesized and natural compounds can be of importance in suppressing DOX resistance via regulating Nrf2 and its downstream targets. In this section, a mechanistic discussion of signaling networks in which Nrf2 is key player and lead to DOX resistance, is provided to provide insights for developing novel therapeutics.

One of the important aspects of Nrf2 signaling is its association with drug transporters. Nrf2 can promote expression of P-gp transporter in enhancing colorectal cancer progression and triggering chemoresistance [206]. Such a relationship is found between Nrf2 and ABCB1 in DOX resistant. In hypoxic conditions, liver cancer cells increase Nrf2 expression to up-regulate activity and expression of ABCB1. Then, intracellular accumulation of DOX decreases in cancer cells that provides their resistance to apoptosis [207]. It has been reported that Nrf2 down-regulation is associated with P-gp inhibition and triggering DOX sensitivity of cancer cells [208]. Targeting Nrf2 is of importance for increasing sensitivity of cancer cells to DOX chemotherapy.

Small interfering RNA (siRNA) is a powerful genetic tool that is extensively applied in targeting molecular pathways and genes responsible for cancer progression. Recent studies have shown that siRNA can be utilized for increasing sensitivity of cancer cells to chemotherapeutic agents such as cisplatin, paclitaxel, docetaxel and so on [209,210,211]. Similarly, siRNA can be used for mediating DOX sensitivity via targeting Nrf2. Down-regulating Nrf2 expression at mRNA and protein levels is performed by siRNA, and its downstream targets such as HO-1 and NQO1 undergo down-regulation, resulting in ROS overgeneration and enhanced sensitivity to DOX chemotherapy [212]. The interesting point is that in vitro and in vivo experiments have confirmed that Nrf2 overexpression is associated with cancer proliferation, survival and chemoresistance. Abrogation of Nrf2 expression results in an increase in DOX sensitivity via enhancing ROS levels and triggering cancer cell death [213].

Cluster of differentiation 44 (CD44) is a glycoprotein and a receptor for extracellular matrix (ECM) components such as hyaluronic acid (HA). This cell-surface glycoprotein is a cancer stem cell (CSC) marker and can undergo alternative splicing and post-transcriptional modification. CD44 overexpression is an obvious finding in cancer cells and mediates their malignancy [214,215]. It has been reported that CD44 can trigger drug resistance of breast cancer stem cells. CD44 enhances p62 expression to induce Nrf2 and DOX resistance [216]. This is maybe due to increased malignancy of cancer cells, so that Keap1 down-regulation and subsequent Nrf2 induction provide conditions for cancer growth [217]. CSCs that are resistant to DOX chemotherapy, demonstrate simultaneous up-regulation of Nrf2 and ABCB1 [218]. As it was mentioned earlier, Nrf2 stimulates DOX resistance via enhancing activity and expression of drug transporters. Therefore, up-regulation of Nrf2 and ABCB1 in CSCs may have associations that should be considered in further experiments.

MRTF-A is a co-activator of serum response factor (SRF) that functions as a tumor-promoting factor in increasing proliferation, and metastasis as well as triggering drug resistance [219]. MRTF-A can cooperate with signal transducer and activator of transcription 3 (STAT3) in inducing BRSM1 hypermethylation and increasing breast cancer invasion [220]. In mediating DOX resistance, MRTF-A generates a complex containing SRF attached to CarG on promoter region of Nrf2 to stimulate its expression and reduce sensitivity of cancer cells to apoptosis [221].

**Figure 3:**
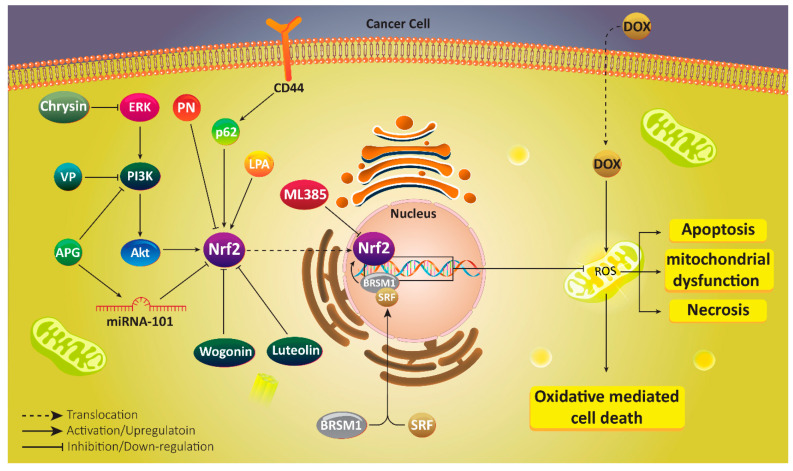
Nrf2 signaling in mediating DOX resistance of cancer cells. Suppressing Nrf2 signaling as a pro-survival pathway is associated with induction of apoptosis and necrosis in cancer cells, and their sensitivity to chemotherapy.

Sometimes, interaction between enzymes and their product can direct conditions towards developing chemoresistance. Such association is obvious in lysophosphatidate (LPA) that is generated by autotaxin (ATX). Primarily, LPA is involved in repairing tissues by inducing proliferation, migration, angiogenesis and other important biological mechanisms. These impacts are of importance in improving pathological conditions such as arthritis, pulmonary fibrosis and inflammatory bowel disease [222,223,224,225,226]. However, it has been reported that LPA and ATX can induce cancer progression and LPA up-regulation is correlated with development of colon cancer and hepatitis [227,228,229]. In DOX-resistant breast cancer cells, LPA enhances stabilization of Nrf2 to up-regulate its expression, resulting in activation of antioxidant parameters and drug transporters that are vital for inducing DOX resistance [230]. Table 2 provides a summary of molecular pathways involved in DOX resistance, and anti-tumor agents capable of regulating Nrf2 signaling in suppressing DOX resistance (Table 2, Figure 3).

## 9. Room for Drug Discovery

In treatment of cancer patients, DOX is considered as a first option and is mostly preferred to surgery, as an invasive strategy. However, resistance to this well-known chemotherapeutic agent has resulted in failure in treatment of cancer patients. Nrf2 is involved in DOX resistance, and after chemotherapy, compounds activating Nrf2 signaling can be applied. As it was mentioned earlier in the main text, most of the anti-tumor compounds for providing DOX sensitivity are phytochemicals. For synthesizing new small molecule inhibitors of Nrf2 signaling, much attention should be directed towards Nrf2 and Keap1 structures. Furthermore, new chemically synthesized anti-tumor agents can also inhibit nuclear translocation of Nrf2 by binding to it and providing ubiquitination and degradation. After DOX chemotherapy, the story is completely different and if a protective agent wants to be synthesized, it should be capable of binding to Nrf2 and mediating its nuclear translocation or suppressing Keap1 activity.

## 10. Conclusions and Remarks

In the present review, two important aspects of Nrf2 signaling including chemoprotection and chemoresistance were discussed in view of DOX. Each section was divided into two parts describing involved molecular pathways and role of anti-tumor and protective compounds in targeting Nrf2 signaling during DOX chemotherapy. It is noteworthy that most of the compounds targeting Nrf2 signaling are phytochemicals. In the case of protecting against adverse effects of DOX, protective compounds induce Nrf2 signaling and its downstream targets such as HO-1 and NQO1 in reinforcing antioxidant defense systems and supporting against oxidative damage, while anti-tumor compounds inhibit Nrf2 signaling in promoting ROS levels and oxidative damage, resulting in cell death in cancer cells.

These statements clearly demonstrate the dual role of Nrf2 signaling in cancer chemotherapy. In fact, the aim is determining factor for stimulating or suppressing Nrf2 signaling. The notion should be considered that chemoprotection should be performed after DOX chemotherapy, since inducing Nrf2 signaling is associated with DOX resistance. Therefore, Nrf2 inhibition should be conducted during DOX chemotherapy and Nrf2 induction after this period to prevent or ameliorate its side effects on major organs of the body. The interesting point is that Nrf2 signaling can promote stem cell population in providing DOX resistance. Hence, by targeting Nrf2 signaling, both cancer cells and CSCs are affected that are of importance in effective DOX chemotherapy.

To date, most of the studies have focused on using compounds for targeting Nrf2 signaling in chemoprotection and reversing chemoresistance. However, more progress can be performed using nanoparticles for delivery of these anti-tumor agents. Nanocarriers can significantly promote intracellular accumulation of anti-tumor agents in cancer cells and enhance their efficiency in Nrf2 inhibition and providing DOX sensitivity. Furthermore, the ability of compounds to protect during DOX chemotherapy can be improved using nanocarriers. Another important aspect is using genetic tools in targeting Nrf2 signaling. As it was mentioned, siRNA system has been applied for affecting Nrf2 signaling. Other techniques such as CRISPR/Cas9 system can also be used, and furthermore, more studies are needed to elucidate potential of siRNA for using in DOX sensitivity. Similar to compounds, nanoarchitectures can promote efficiency of genetic tools in gene silencing that should be considered in future experiments.

## Figures and Tables

**Table 1:** Nrf2 signaling as a chemoprotection mechanism.

Toxicity	Signaling Network	Compound	Nrf2 Expression	Outcomes	Refs
**Cardiotoxicity**	MiRNA-140-5p/Nrf2	–	Down-regulation	Deteriorating DOX-mediated cardiotoxicity Reducing expressions of NQO1 and HO-1 Enhancing oxidative stress level	[140]
**Cardiotoxicity** **Hepatotoxicity** **Renotoxicity**	–	Asiatic acid	Up-regulation	Reducing necrosis, congestion and hyaline degeneration in heart Decreasing leukocyte inflammation, necrosis, apoptosis and fatty change in liver Decreasing necrosis and inflammation in kidney Mediating these protective effects via Nrf2 induction	[98]
**Cardiotoxicity**	Nrf2/HO-1 Nrf2/NQO1 Nrf2/GCL	Pristimerin	Up-regulation	Increasing expressions of Nrf2 and its downstream targets HO-1, NQO1 and GCL Reducing oxidative stress and fibrosis	[91]
**Cardiotoxicity**	Nrf2/HO-1 Nrf2/NQO1	Tert-butylhydroquinone	Up-regulation	Ameliorating cardiotoxicity via induction of Nrf2 and its downstream targets	[113]
**Cardiotoxicity**	Nrf2/HO-1	b-LAPachone	Up-regulation	Triggering nuclear translocation of Nrf2 Enhancing expressions of HO-1 and antioxidant enzymes such as SOD, CAT and GPx	[101]
**Cardiotoxicity**	Nrf2/HO-1 Nrf2/NQO1	Cardamonin	Up-regulation	Protecting cells against inflammation and oxidative stress Reducing oxidative stress, apoptosis, and inflammation Inducing Nrf2 signaling and its downstream targets HO-1 and NQO1	[105]
**Cardiotoxicity**	Nrf2/HO-1	Curdione	Up-regulation	Alleviating oxidative stress Preventing ROS overgeneration and mediating mitochondrial dysfunction Triggering Nrf2/HO-1 axis as an antioxidant axis	[102]
**Cardiotoxicity**	MAPK/Nrf2/ARE	Chitosan oligosaccharide	Up-regulation	Decreasing oxidative stress and apoptosis Stimulating MAPK and subsequent induction of Nrf2/ARE axis Reinforcing antioxidant defense system	[112]
**Cardiotoxicity**	MiRNA-200a/Nrf2	–	Up-regulation	Improving cardiomyocyte contractile function Reducing levels of cardiac troponin I Ameliorating oxidative stress, inflammation and apoptosis Inducing Nrf2 signaling	[143]
**Cardiotoxicity**	Nrf2/ARE	3,3′-diindolylmethane	Up-regulation	Suppressing apoptosis Improving histopathological profile Enhancing expressions of HO-1, NQO1 and GST Reducing Bax and caspase-3 expression	[114]
**Cardiotoxicity**	Sirt1/AMPK/Nrf2	Acacetin	Up-regulation	Alleviation of cardiomyopathy Enhancing cell viability Preventing ROS overgeneration Activation of Sirt1/AMPK to induce Nrf2 signaling Triggering cell defense system	[170]
**Cardiotoxicity**	Nrf2/HO-1	Genistein	Up-regulation	Inducing Nrf2/HO-1 axis Reducing ROS levels by its scavenging feature Reducing lipid peroxidation and DNA damage	[171]
**Cardiotoxicity**	Nrf2/LC-3II/autophagy	–	Down-regulation	Reducing oxidative stress Activating autophagy as a protective mechanism via LC-3II up-regulation Nrf2 inhibition aggravates DOX-mediated cardiotoxicity via impairing autophagy and enhancing oxidative stress	[144]
**Cardiotoxicity**	–	p-coumaric acid	Up-regulation	Enhancing cell survival Inhibiting apoptosis and oxidative stress Providing nuclear translocation of Nrf2	[118]
**Cardiotoxicity**	Nrf2/NQO1	Tanshinone IIA	Up-regulation	Enhancing cell viability and morphological profile Reducing oxidative parameters Up-regulation of NQO1	[124]
**Cardiotoxicity**	ORM1/Nrf2	–	Up-regulation	ORM1 is correlated with a decrease in oxidative stress and apoptosis Up-regulation of Nrf2 and its downstream target HO-1	[158]
**Testicular toxicity**	–	–	Down-regulation	Inducing apoptosis and oxidative stress in testis Reducing Nrf2 expression	[149]
**Nephrotoxicity**	–	Thymoquinone	Up-regulation	Reducing malondialdehyde and lipid peroxidation levels Enhancing SOD and GST levels Preventing necrosis and oxidative stressActivation of Nrf2 and improving antioxidant defense system	[119]

**Table 2:** Activation/suppression of Nrf2 signaling and its association with DOX resistance/sensitivity.

Cancer Type	Signaling Network	Compound/Agent	Nrf2 Expression	Remarks	Refs
**Breast cancer**	P62/Nrf2	–	Up-regulation	Reducing oxidative stress Mediating DOX resistance Promoting colony formation and migration capacities Improving cancer stem cell features	[216]
**Breast cancer**	Cul3/Nrf2	–	Down-regulation	Association of Cul3 with Nrf2 depletion Inducing oxidative stress Increasing DOX sensitivity	[231]
**Breast cancer**	PI3K/Nrf2/MRP1	Vielanin P	Down-regulation	Inhibiting PI3K/Nrf2 axis Suppressing MRP1 expression Promoting DOX sensitivity	[182]
**Breast cancer**	Nrf2/HSP70	Parthenolide	Down-regulation	Reducing expressions of Nrf2 and HSP70 Enhancing DOX sensitivity of breast cancer cells	[176]
**Breast cancer**	Nrf2/HO-1 Nrf2/MDR1	Luteolin	Down-regulation	Enhancing number of cancer cells undergoing cell death Increasing cytotoxicity of DOX Down-regulation of Nrf2 and subsequent inhibition of its downstream targets HO-1 and MDR1	[202]
**Breast cancer**	Nrf2/HO-1 Nrf2/NQO1	Wogonin	Down-regulation	Impairing cellular defense systemNrf2 signaling inhibition Down-regulation of HO-1 and NQO1 Increasing DOX cytotoxicity towards cancer cells	[195]
**Breast cancer**	HER2/Nrf2	–	Up-regulation	Conferring drug resistance Enhancing activities of antioxidant enzymes such as GSTA2, GSTP1 and HO-1	[232]
**Breast cancer**	Nrf2/p62	Pseudomonas aeruginosa mannose-sensitive hemagglutinin	Down-regulation	Inhibiting Nrf2 signaling and its downstream target p62 Increasing DOX sensitivity Impairing cancer growth	[190]
**Hepatocellular carcinoma**	MiRNA-101/Nrf2	Apigenin	Down-regulation	Enhancing miRNA-101 expression Inhibiting Nrf2 signaling by binding to 3^/^-UTR Enhancing DOX sensitivity	[186]
**Hepatocellular carcinoma**	PI3K/Akt/Nrf2	Apigenin	Down-regulation	Reducing mRNA and protein levels of Nrf2 via PI3K/Akt inhibition Reducing cell proliferation Inducing apoptosis Promoting DOX sensitivity	[189]
**Liver cancer**	Nrf2/ABCB1	–	Up-regulation	Nrf2 overexpression occurs in hypoxic conditions Reducing apoptosis and DNA damage Inducing DOX resistance ABCB1 up-regulation	[207]
**Different cancers**	MRTF-A/Nrf2	–	Up-regulation	Reducing apoptosis Triggering DOX resistance	[221]
**Different cancers**	–	–	Down-regulation	SiRNA is a powerful in Nrf2 down-regulation Inhibiting activities of ABCC3, ABCC4 and ABCG2 Enhancing DOX sensitivity	[212]
**Different cancers**	PI3K/Akt/Nrf2 ERK/Nrf2	Chrysin	Down-regulation	Suppressing PI3K/Akt/Nrf2 and ERK/Nrf2 signaling pathwaysNrf2 down-regulation and inhibiting its downstream targets HO-1 Enhancing DOX sensitivity	[181]
**Ovarian cancer**	ALDH/Nrf2	All-trans retinoic acid	Down-regulation	Promoting cancer stem features Enhancing colony formation capacity Mediating DOX resistance Suppressing ALDH/Nrf2 signaling by retinoic acid in reducing DOX resistance	[191]
**Ovarian cancer**	–	–	Up-regulation	Overexpression of Nrf2 in DOX-resistant cancer cells Reducing tumor growth following Nrf2 down-regulation	[233]
**Ovarian cancer**	–	–	Up-regulation	Obtaining DOX resistance via Nrf2 signaling and reducing cell death	[234]
**Ovarian cancer**	Nrf2/miRNA-206/c-MET/EGFR	–	Up-regulation	Reducing miRNA-206 expression Inducing expressions of c-MET and EGFR expressions Triggering DOX resistance	[235]
**Colorectal cancer**	Nrf2/P-gp	–	Up-regulation	Enhancing P-gp expressions Reducing cell death Inducing DOX resistance	[208]
**Lung cancer**	–	ML385	Down-regulation	ML385 functions as an inhibitor of Nrf2 signaling Promoting DOX sensitivity	[172]
**Myeloid leukemia**	Nrf2/HO-1 Nrf2/NQO1	Tritolide	Down-regulation	Enhancing drug sensitivity Apoptosis induction Suppressing Nrf2 and its downstream targets	[203]

## Abbreviations

DOX	Doxorubicin
ROS	reactive oxygen species
TFAM	mitochondrial transcription factor A
AMPK	AMP-activated protein kinase
VASH2	vasohibin2
SpK1	sphingosine kinase 1
HIF-1α	hypoxia inducible factor-1α
ncRNAs	non-coding RNAs
lncRNAs	long non-coding RNAs
miRNAs	microRNAs
GI	gastrointestinal
MMP-2	matrix metalloproteinase-2
Nrf2	nuclear factor erythroid 2-related factor 2
Keap1	kelch-like ECH-associated protein 1
Cul3	cullin3
GSK-3β	glycogen synthase-kinase 3β
βTrCP	β transducing repeat containing E3 ubiquitin-protein ligase
HO-1	heme oxygenase-1
NQO1	NAD(p)H dehydrogenase quinone 1
ARE	antioxidant response elemento
PPARγ	peroxisome proliferator-activated receptor gama
MDA	malondialdehyde
SOD	superoxide dismutase
CAT	catálase
GSH	glutathione
Pris	pristimerin
AA	Asiatic acid
B-LAP	β-LAPachone
CAR	cardamonin
COS	chitosan oligosaccharide
*p*CA	*p*-coumaric acid
LPS	lipopolysaccharide
Tan IIA	tanshinone IIA
PUN	punicalagin
3^/^-UTR	3^/^-untranslated region
LC-3II	light chain-3II
ORM1	orosomucoid 1
3D	3-dimensional
PN	parthenolide
P-gp	P-glycoprotein
HSP70	heat shock protein 70
PI3K	phosphatidylinositide 3-kinase
Akt	protein kinase-B
ERK	extracellular signal-regulated kinase
VP	vialenin P
MRP1	multidrug resistance protein 1
APG	apigenin
EMT	epithelial-to-mesenchymal transition
siRNA	small interfering RNA
CD44	cluster of differentiation 44
ECM	extracellular matrix
CSC	cancer stem cell
SRF	serum response factor
STAT3	signal transducer and activator of transcription 3
LPA	lisophosphatidate
ATX	autotaxin
